# Autopsy‐Confirmed CMV Adrenalitis With Bilateral Adrenal Hemorrhage Following Presumed Critical Illness–Related Corticosteroid Insufficiency During Prolonged ICU Stay: A Case Report

**DOI:** 10.1155/crcc/6331364

**Published:** 2026-05-25

**Authors:** Yoko Kanamitsu, Yusuke Seino, Natsumi Toyoda, Yoshiya Sugiura, Tomonori Takano, Hiroyuki Kunishima, Yukio Sato, Masaki Izumo, Shigeki Fujitani

**Affiliations:** ^1^ Department of Emergency and Critical Care Medicine, St. Marianna University School of Medicine, Kawasaki, Kanagawa, Japan, marianna-u.ac.jp; ^2^ Department of Anesthesiology, St. Marianna University School of Medicine, Kawasaki, Kanagawa, Japan, marianna-u.ac.jp; ^3^ Department of Pathology, St. Marianna University School of Medicine, Kawasaki, Kanagawa, Japan, marianna-u.ac.jp; ^4^ Department of Infectious Diseases, St. Marianna University School of Medicine, Kawasaki, Kanagawa, Japan, marianna-u.ac.jp; ^5^ Department of Cardiology, St. Marianna University School of Medicine, Kawasaki, Kanagawa, Japan, marianna-u.ac.jp

**Keywords:** adrenal hemorrhage, adrenal insufficiency, adrenalitis, critical illness–related corticosteroid insufficiency, cytomegalovirus

## Abstract

**Background:**

In critically ill patients, critical illness–related corticosteroid insufficiency (CIRCI) is an important cause of vasopressor‐refractory shock. However, distinguishing relative or functional CIRCI from structural adrenal insufficiency—such as that caused by hemorrhagic adrenal necrosis—can be challenging because their clinical presentations often overlap despite fundamentally different pathophysiological mechanisms. Cytomegalovirus (CMV) adrenalitis with hemorrhagic adrenal necrosis is rare, potentially fatal, and may be overlooked.

**Case Presentation:**

A 70‐year‐old man with ischemic cardiomyopathy and diabetic nephropathy–related chronic kidney disease, without known immunodeficiency, was admitted for acute decompensated right‐sided heart failure. He required intensive care and continuous renal replacement therapy for refractory right‐sided heart failure and renal failure. During his ICU stay, he experienced two episodes of vasopressor‐refractory shock. Although the random serum cortisol level was not unequivocally low, hydrocortisone was administered for clinically presumed CIRCI, resulting in transient hemodynamic improvement. After tapering and discontinuation of hydrocortisone, shock recurred and progressed despite treatment for presumed septic shock, and the patient died. Autopsy revealed extensive bilateral hemorrhagic adrenal necrosis. Microscopic examination demonstrated characteristic “owl′s eye” intranuclear inclusions and positive CMV immunohistochemistry, consistent with CMV adrenalitis causing acute structural adrenal failure.

**Conclusion:**

Even in critically ill patients without apparent baseline immunodeficiency, secondary immunosuppression due to prolonged ICU care may facilitate CMV reactivation, leading to fatal adrenal insufficiency. This case highlights the importance of distinguishing presumed CIRCI from evolving structural adrenal failure and of considering CMV adrenalitis in prolonged ICU patients with recurrent shock.

## 1. Introduction

In the ICU, vasopressor‐refractory shock is life‐threatening and demands rapid etiologic evaluation and timely intervention. One contributor is the endocrine maladaptation termed critical illness–related corticosteroid insufficiency (CIRCI), for which low‐dose glucocorticoid therapy may improve hemodynamics in septic shock. However, CIRCI lacks universally accepted diagnostic thresholds; random cortisol levels and ACTH stimulation testing have limitations [1, 2]. As a result, distinguishing functional CIRCI from acute adrenal insufficiency due to structural adrenal disease is challenging in practice [1]. CMV adrenalitis is a rare but potentially fatal cause of structural adrenal insufficiency and may be difficult to recognize during prolonged critical illness, particularly when the clinical presentation overlaps with CIRCI. Reports of CMV adrenalitis with bilateral adrenal hemorrhage in patients without known baseline immunodeficiency remain scarce. We report an autopsy‐confirmed case in which shock initially managed as suspected CIRCI was followed by fatal CMV adrenalitis with bilateral hemorrhagic adrenal necrosis during prolonged ICU care.

## 2. Case Presentation

A 70‐year‐old man with advanced ischemic cardiomyopathy–related chronic heart failure (left ventricular ejection fraction of approximately 30% with right ventricular dysfunction, consistent with end‐stage heart failure) and chronic kidney disease due to diabetic nephropathy, representing advanced cardiorenal disease, was admitted to our hospital; his HbA1c on admission was 7.5%. He had no known primary immunodeficiency and no exposure to immunosuppressants prior to admission. He presented with weight gain and bilateral leg edema and was admitted for decompensated right‐sided heart failure. Despite inotropes and diuretics, congestion and hypotension persisted. Two months after admission, he was transferred to the ICU, and continuous renal replacement therapy was initiated for fluid management.

On ICU Day 10, he developed hypotension with rigors and fever, and septic shock was suspected. Broad‐spectrum antimicrobials and hemodynamic support were started. The next day, he suffered from cardiac arrest immediately after repositioning and underwent venoarterial extracorporeal membrane oxygenation (VA‐ECMO). Because intra‐abdominal hypertension with abdominal distension was suspected, large‐volume serous ascites was drained, after which hemodynamics improved, and VA‐ECMO was discontinued on ICU Day 15. Blood, urine, and ascitic fluid cultures remained negative. Consciousness impairment persisted after weaning from VA‐ECMO, requiring prolonged mechanical ventilation.

**Figure 1:**
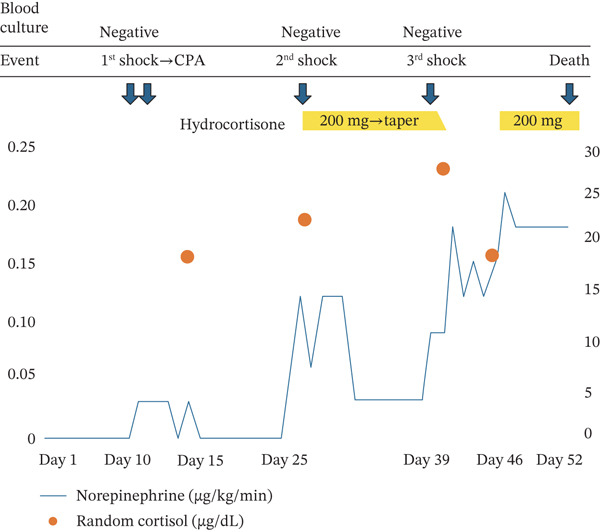
Timeline of treatment and hemodynamic changes. Arrows indicate clinical events, and yellow bars represent hydrocortisone administration (200 mg/day followed by tapering after the second shock episode and 200 mg/day after the third shock episode). The graph illustrates the norepinephrine dose (solid blue line, micrograms per kilogram per minute) and random serum cortisol levels (orange dots, micrograms per deciliter). Note the marked decrease in vasopressor requirement immediately following hydrocortisone initiation on ICU Day 25. Abbreviation: CPA, cardiopulmonary arrest.

On ICU Day 25, he again developed fever and hypotension. Despite increased vasopressors, improvement was insufficient, and septic shock was suspected. Although the random serum cortisol level was 22.3 *μ*g/dL and not unequivocally low, CIRCI could not be ruled out clinically; therefore, intravenous hydrocortisone (200 mg/day) was initiated, with prompt blood pressure improvement allowing vasopressor reduction. At that time, blood and urine cultures were negative, and there was no hypoglycemia, hyponatremia, hyperkalemia, or eosinophilia. As his overall status stabilized, hydrocortisone was tapered and discontinued. The day after discontinuation (ICU Day 39), he developed fever; a culture of the removed central venous catheter tip yielded coagulase‐negative staphylococci, and catheter‐related bloodstream infection was suspected and managed by catheter removal and targeted antibiotics. On ICU Day 46, a third shock episode occurred. He received fluid resuscitation, vasopressors, and antibiotics for presumed septic shock. Hydrocortisone was reinitiated for possible recurrent CIRCI; however, his hemodynamics failed to improve, and the shock state progressed. A computed tomography (CT) scan performed on ICU Day 48 revealed no apparent abnormalities, and, in particular, neither adrenal enlargement nor hemorrhagic lesions were identified. Despite multidisciplinary intensive care, the circulatory failure was irreversible, and he died on ICU Day 52 (Figure 1).

**Figure 2:**
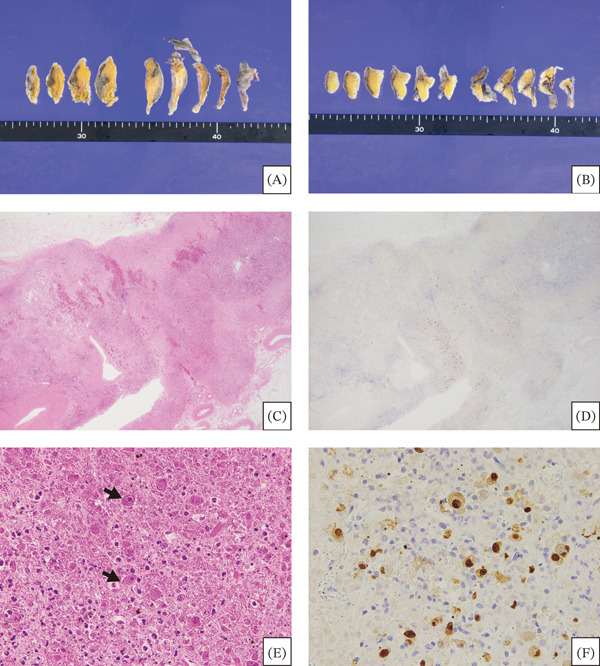
Autopsy findings of the adrenal glands. (A, B) Macroscopic appearance of the cut surfaces of the (A) right and (B) left adrenal glands. Normal yellow cortical tissue is sparingly preserved, replaced by extensive bilateral dark‐red hemorrhagic necrosis. (C, D) Low‐power fields. (C) Hematoxylin and eosin (H&E) staining shows destruction of the corticomedullary architecture with extensive coagulative necrosis and hemorrhage. (D) Immunohistochemistry for CMV antigen shows extensive positivity. (E, F) High‐power fields. (E) H&E staining reveals a loss of normal adrenal cells and the presence of numerous cytomegalic cells with characteristic “owl′s eye” intranuclear inclusions (arrows). (F) These cytomegalic cells are positive for CMV antigen.

Pathologic examination showed extensive coagulative necrosis and hemorrhage in the bilateral adrenal glands. Notably, cells with characteristic “owl′s eye” intranuclear inclusions were scattered throughout the cortex and medulla, and immunohistochemically, these cells were positive for CMV antigen (Figure 2). CMV‐infected cells were also scattered in the bilateral lung and liver without overt histological damage. These findings were compatible with CMV adrenalitis with bilateral hemorrhagic adrenal necrosis causing acute adrenal insufficiency.

## 3. Discussion

This case illustrates that in prolonged critical illness, secondary immunosuppression may enable CMV reactivation with adrenal tropism, culminating in fatal adrenal failure due to CMV adrenalitis with bilateral hemorrhagic adrenal necrosis despite transient responsiveness to corticosteroids.

CMV adrenalitis has been reported predominantly in patients with acquired immunodeficiency syndrome, recipients of solid organ or hematopoietic stem cell transplantation, hematologic malignancies, or those receiving intensive immunosuppression. Sporadic cases in apparently immunocompetent hosts are exceedingly rare; to our knowledge, only a single surgical case has been described. In that report, surveillance fluorodeoxyglucose (FDG)–positron emission tomography CT in a woman with a remote history of breast cancer revealed an FDG‐avid adrenal mass initially presumed to represent metastatic disease; however, adrenalectomy demonstrated CMV adrenalitis in the absence of shock or adrenal failure, and adrenal hormone levels were not systematically evaluated [3].

In this patient, the measured random serum cortisol value was not markedly low; however, because CIRCI could not be excluded clinically in the setting of vasopressor‐refractory shock, empiric hydrocortisone was initiated. Ultimately, autopsy disclosed bilateral hemorrhagic CMV adrenalitis with adrenal failure. More broadly, contemporary CIRCI guidelines focus on functional hypothalamic–pituitary–adrenal axis dysfunction and offer limited guidance on distinguishing CIRCI from structural adrenal disease (e.g., CMV adrenalitis or hemorrhagic infarction). Random cortisol measurements have limited discriminatory value in critical illness, proposed thresholds for these measurements vary widely, and even values above the cutoffs may fail to identify patients who are clinically judged to have impaired adrenal reserve. Moreover, the typical biochemical hallmarks of primary adrenal insufficiency (hyponatremia, hyperkalemia, eosinophilia, and low cortisol with high ACTH) may be obscured by fluid resuscitation, renal replacement therapy, or concomitant medications [4, 5]. Imaging also has important limitations: Although established hemorrhage often yields adrenal enlargement or high‐attenuation lesions on CT/magnetic resonance imaging (MRI), early bilateral adrenal hemorrhage can be radiologically occult; several reports describe normal early abdominal CT with abnormalities emerging only on repeat imaging days later or being recognized solely at autopsy [6, 7]. Consistent with these pitfalls, a contrast‐enhanced CT on ICU Day 48 (4 days prior to death) showed no adrenal abnormality, whereas autopsy revealed extensive bilateral hemorrhagic CMV adrenalitis. Taken together, these findings underscore that structural adrenal disease should remain in the differential diagnosis even when serum cortisol levels are not markedly low and CT findings do not show obvious adrenal abnormalities.

In our patient, prolonged ICU stay, persistent inflammation, hypoalbuminemia, and lymphopenia suggested progression toward chronic critical illness (CCI) and the persistent inflammation–immunosuppression–catabolism syndrome (PICS) phenotype [8–10]. In CCI/PICS, secondary immunodeficiency predisposes not only to bacterial and fungal infections but also to reactivation of latent viruses, including CMV [10, 11]. Observational studies in inflammatory bowel disease and rheumatic disease cohorts indicate that systemic corticosteroids and hypoalbuminemia are associated with CMV infection/reactivation risk [12–16] and hypoalbuminemia was also identified as a risk factor for CMV reactivation in patients with ANCA‐associated vasculitis (J‐CANVAS) [17]. Our patient received a cumulative hydrocortisone dose of approximately 2300 mg as part of CIRCI‐directed therapy. This exposure combined with chronic inflammation and malnutrition may have contributed to CMV reactivation and adrenal involvement. In our case, the presence of pathognomonic “owl′s eye” inclusions within the necrotic tissue provided definitive evidence of CMV pathogenicity, distinguishing it from mere bystander reactivation.

When initiating corticosteroids for presumed CIRCI in patients with prolonged ICU stays, clinicians should (i) recognize heightened CMV reactivation risk in CCI/PICS, (ii) consider CMV testing (antigenemia or polymerase chain reaction) when fever or shock lacks an identifiable source, (iii) acknowledge that the absence of adrenal enlargement or hemorrhage on imaging does not exclude evolving structural adrenal disease, including bilateral adrenal hemorrhage, and (iv) consider early antiviral therapy if CMV adrenalitis is suspected, alongside steroid supplementation. Systematic screening for viral reactivation and heightened vigilance for adrenal pathology may improve detection and outcomes in this population.

Because CMV‐specific IgG and IgM levels were not measured, we could not definitively distinguish primary infection from reactivation in this case. However, CMV seroprevalence among Japanese adults exceeds 70% and increases further with age, making prior latent CMV infection highly probable in this patient [18]. Taken together with his prolonged critical illness, secondary immunosuppression, and the characteristic histopathological features of CMV adrenalitis, reactivation of latent CMV is a more plausible explanation than de novo primary infection in this patient.

## 4. Conclusion

Even in the absence of pre‐existing immunodeficiency, prolonged critical illness can permit CMV adrenalitis with bilateral hemorrhagic adrenal necrosis. In patients with recurrent vasopressor‐refractory shock during prolonged ICU care, clinicians should distinguish CIRCI from evolving structural adrenal failure and consider the possibility of CMV adrenalitis, with targeted virologic testing, and repeat imaging evaluation as clinically indicated.

## Author Contributions

Yoko Kanamitsu: conceptualization, investigation, writing—original draft, writing—review and editing, and visualization; Yusuke Seino and Shigeki Fujitani: supervision, conceptualization, and writing—review and editing; Natsumi Toyoda and Yoshiya Sugiura (pathology): investigation, resources, and writing—review and editing; Tomonori Takano and Hiroyuki Kunishima (infectious diseases): conceptualization and writing—review and editing; Yukio Sato and Masaki Izumo: supervision (clinical supervision of patient management).

## Funding

No funding was received for this manuscript.

## Disclosure

All authors approved the final manuscript and agree to be accountable for all aspects of the work.

## Ethics Statement

Ethical review was not required for this study because it is a single‐patient case report in accordance with institutional policy.

## Consent

Written informed consent was obtained from the patient′s family for the publication of this case report and any accompanying images.

## Conflicts of Interest

The authors declare no conflicts of interest.

## Data Availability

Deidentified clinical data underlying this report are available from the corresponding author upon reasonable request.
